# Evaluating the PM ACTIVAS Nurse-Led Preventive Care Model for Community-Dwelling Older Adults: A Randomized Controlled Trial

**DOI:** 10.3390/healthcare14193151

**Published:** 2026-09-23

**Authors:** Claudia Bustamante-Troncoso, Francisca Marquez-Doren, Claudia Alcayaga-Rojas, Víctor Pedrero, Miriam Rubio-Acuña, Marcela González-Agüero, Patricia Manns-Gantz, Solange Campos-Romero, Hugo Sánchez-Reyes, Ignacio Ibarra-Torres, Claudia Valderrama-Ulloa, Camila Lucchini-Raies

**Affiliations:** 1 School of Nursing, Pontificia Universidad Católica de Chile, Santiago 7820436, Chile; cqbustam@uc.cl (C.B.-T.); caalcaya@uc.cl (C.A.-R.); merubioa@uc.cl (M.R.-A.); mmgonzal@uc.cl (M.G.-A.); scamposr@uc.cl (S.C.-R.); igibarra@uc.cl (I.I.-T.); 2 Alpha Beta Omicron Chapter Sigma International, Santiago 7820436, Chile; 3 Faculty of Nursing, Universidad Andrés Bello, Santiago 8370136, Chile; victor.pedrero@unab.cl; 4 School of Design, Pontificia Universidad Católica de Chile, Santiago 7510596, Chile; pmanns@uc.cl; 5 Hospital Padre Hurtado, Santiago 9710000, Chile; hugo.sanchez@hurtadohosp.cl; 6 Center for Research in Technologies for Society, Faculty of Engineering, Universidad del Desarrollo, Santiago 7610658, Chile; claudia.valderrama@udd.cl

**Keywords:** accidental falls, primary health care, home visits, healthy ageing, nurse-led care, community-dwelling older adults

## Abstract

**Highlights:**

**What are the main findings?**

- The PM ACTIVAS nurse-led preventive care model did not significantly reduce the number of falls (primary outcome) or fall occurrence (secondary outcome) during the 12-month follow-up.
- The intervention reduced modifiable fall-risk factors and improved participants’ recognition of environmental fall hazards compared with usual care.

**What are the implications of the main findings?**

- Nurse-led preventive care models may provide a structured approach to operationalizing evidence-based fall prevention within routine primary health care.
- PM ACTIVAS provides a structured framework that could be adapted and evaluated in other community-based primary health care settings.

**Abstract:**

Background/Objectives: Falls among community-dwelling older adults are a major public health challenge and a leading cause of functional decline. Although multifactorial fall prevention strategies are recommended, evidence on comprehensive nurse-led preventive care models in primary health care remains limited. This study evaluated the clinical effects of the PM ACTIVAS nurse-led preventive care model among community-dwelling older adults in routine primary health care. Methods: This article reports the quantitative component of a broader mixed-methods research programme. A two-arm randomized controlled trial included 220 community-dwelling adults aged 65–80 years randomly assigned to the PM ACTIVAS intervention (n = 110) or usual care (n = 110). The 12-month intervention combined multidimensional fall-risk assessment, individualized care planning, home environmental assessment, health education, a home safety kit, family involvement, and monthly telephone follow-up. The primary outcome was the number of falls (fall frequency) during the 12-month follow-up; secondary outcomes were fall occurrence, modifiable fall-risk factors, recognition of environmental fall hazards, and fear of falling. Results: Primary outcome data were available for 145 participants, with no significant between-group difference in the number of falls (*p* = 0.164). Fall occurrence, a secondary outcome, also did not differ significantly between groups (17 participants [23.3%] in the intervention group vs. 19 [26.4%] in the control group; *p* = 0.666). After adjustment for baseline scores, the intervention group showed significantly fewer fall-risk factors (*p* = 0.001; partial η^2^ = 0.07) and greater recognition of environmental fall hazards (*p* = 0.044; partial η^2^ = 0.02). No between-group differences were observed in fear of falling. Conclusions: Although the PM ACTIVAS nurse-led preventive care model did not significantly reduce the number of falls over 12 months, it reduced modifiable fall-risk factors and improved recognition of environmental hazards. These findings suggest that coordinated nurse-led care may provide a structured approach to operationalizing evidence-based fall prevention within routine primary health care and support further evaluation of this model in community settings.

## 1. Introduction

Maintaining functional ability has become one of the principal goals of health systems caring for rapidly ageing populations. The World Health Organization (WHO) defines healthy ageing as the process of developing and maintaining the functional ability that enables well-being in older age, shifting the focus of care from disease treatment toward preserving intrinsic capacity, autonomy, and quality of life [1,2]. This paradigm recognizes that enabling older adults to remain safely in their own homes and communities (“ageing in place”) requires proactive, person-centred, and community-based models of care that identify modifiable risks before disability occurs. Consequently, preventive care has become a strategic priority for primary health care [3], positioning nurses to lead comprehensive interventions that promote healthy ageing and maintain independence [1,4].

Falls represent one of the greatest threats to healthy ageing because they compromise not only physical health but also functional independence, confidence, and social participation. Approximately one in four community-dwelling older adults experiences at least one fall annually, making falls one of the leading causes of injury, disability, hospitalization, institutionalization, and mortality worldwide [4,5]. Beyond their physical consequences, falls frequently trigger fear of falling, reduced mobility, social isolation, and progressive loss of functional capacity, often initiating a cycle of dependency that substantially affects quality of life and healthcare utilization [6]. Current evidence recognizes that falls rarely result from a single cause; instead, they emerge from complex interactions among intrinsic factors, medications, behaviors, environmental hazards, and social circumstances that evolve over time [4]. Preventing falls, therefore, should be understood as part of a broader strategy to preserve functional ability and support healthy ageing.

The growing recognition of this complexity has transformed fall prevention strategies over the past decade. International clinical guidelines now recommend comprehensive, multidimensional assessment followed by individualized interventions that address each older person’s specific risk profile [4,7]. Although exercise remains the intervention supported by the strongest evidence, systematic reviews and recent clinical recommendations indicate that combining complementary strategies—including home-hazard assessment and modification, medication review, education, behavioral counselling, and individualized care planning—can provide greater benefits for selected older adults [5,6,8,9,10]. However, important debate remains regarding the relative contribution of each intervention component and the populations most likely to benefit from multifactorial approaches. Increasingly, researchers argue that successful prevention depends not only on the intervention components themselves but also on the extent to which care is individualized, acceptable to older adults, and responsive to their living context and preferences [11,12]. These observations suggest that the challenge is no longer simply identifying effective intervention components but delivering them in a coordinated and sustainable manner within routine primary health care.

Although multifactorial interventions are increasingly recommended, evidence regarding comprehensive nurse-led preventive care models capable of operationalizing evidence-based fall prevention within routine primary health care remains limited, particularly in Latin America [13,14,15]. Most randomized trials have evaluated isolated intervention components, whereas fewer studies have examined integrated nurse-led models of preventive care that combine multidimensional assessment, individualized education, environmental modification, family involvement, and longitudinal follow-up within primary health care [13,16]. In this context, an important gap concerns not only which fall-prevention components are effective, but also how these components can be systematically coordinated, individualized, and sustained within routine primary health care through a clearly defined care delivery model.

The PM ACTIVAS Program was developed in Chile to address this gap as a structured nurse-led preventive care model in which multidimensional fall-risk assessment is translated into individualized care planning and coordinated with home environmental modification, health education, family involvement, and longitudinal follow-up within routine primary health care. Rather than introducing entirely new fall-prevention components, PM ACTIVAS is distinguished by the way these evidence-based strategies are coordinated and individualized within a structured nurse-led care pathway. This approach extends fall prevention beyond the identification of individual risk factors by embedding preventive care within the older person’s everyday living context and routine primary health care. The intervention protocol was previously published [17], followed by a qualitative evaluation demonstrating its feasibility and acceptability among older adults and healthcare professionals [18]. This evaluation showed that older adults particularly valued the home-based and individualized nature of the intervention, the continuity provided through longitudinal nurse follow-up, and recommendations tailored to their everyday living conditions. The trusting relationship established between older adults and nurse-interventionists also emerged as a central feature of the intervention, while participants perceived the agreed preventive recommendations as achievable, clearly defined, and relevant to their needs [18]. The present study reports the final phase of this programme, evaluating the model’s clinical effects through a randomized controlled trial.

Generating evidence on effective and implementable nurse-led preventive care models is essential to strengthen primary health care responses to population ageing. Accordingly, advancing fall prevention requires not only identifying effective intervention components but also developing care models capable of operationalizing this evidence within routine primary health care. Therefore, the aim of this randomized controlled trial was to evaluate the clinical effectiveness of the PM ACTIVAS nurse-led preventive care model among community-dwelling older adults, with the number of falls during the 12-month follow-up as the primary outcome, and to examine its effects on fall occurrence, modifiable fall-risk factors, recognition of environmental fall hazards, and fear of falling as secondary outcomes. We hypothesized that participants receiving the PM ACTIVAS intervention would experience fewer falls during the 12-month follow-up than those receiving usual primary care.

## 2. Materials and Methods

### 2.1. Study Design

This study reports the quantitative component of a broader mixed-methods research programme designed to develop, implement, and evaluate the PM ACTIVAS model, specifically the findings of a two-arm randomized controlled trial conducted to evaluate the clinical effects of the intervention. The qualitative evaluation of feasibility, acceptability, and implementation experiences has been reported separately [18]. The overall programme followed the Medical Research Council framework for the development and evaluation of complex interventions [19].

The trial was conducted between January 2021 and April 2022 in primary health care centres in the municipality of Renca, Santiago, Chile. The study protocol was prospectively registered at ClinicalTrials.gov (https://clinicaltrials.gov/study/NCT04313062; accessed on 16 March 2020) and has been published previously, including the theoretical rationale, intervention development, implementation strategy, and complete intervention description according to the Template for Intervention Description and Replication (TIDieR) checklist [17]. Therefore, only a brief description of the intervention is presented here. The study is reported in accordance with the Consolidated Standards of Reporting Trials (CONSORT) guidelines (Supplementary Table S1) [20].

### 2.2. Study Setting and Participants

Participants were recruited from the ‘Más Adultos Mayores Autovalentes’ Program implemented in primary health care centres. Community-dwelling older adults aged 65–80 years who were functionally independent and had completed the national preventive health assessment within the previous 12 months were considered eligible. Functional independence was operationalized using Part A of the Functional Assessment of Older Adults (Evaluación Funcional del Adulto Mayor [EFAM-Chile]), which forms part of the national preventive health assessment for older adults (EMPAM). EFAM-A was administered during eligibility assessment; participants scoring ≥ 43 points were classified as functionally independent and were eligible for inclusion, whereas those scoring ≤ 42 points were classified as being at risk of dependency and were excluded.

Individuals presenting severe visual or hearing impairment preventing effective communication, cognitive or mood-related conditions identified during the standardized eligibility assessment, hospitalization during recruitment, or residence outside the participating health centres’ catchment area were excluded. Cognitive and mood-related eligibility was assessed using the corresponding components of EFAM Part B, which includes cognitive screening using the Mini-Mental State Examination (MMSE) and questions addressing depressive mood and anxiety symptoms.

Following completion of the baseline assessment, participants were randomly allocated in a 1:1 ratio to the intervention or control group using simple randomization based on a computer-generated random number sequence. The allocation sequence was generated by a researcher who was not involved in participant recruitment, eligibility assessment, or intervention delivery. Allocation concealment was maintained using sequentially numbered, opaque, sealed envelopes. These envelopes were kept exclusively by the fieldwork coordinator, who was the only person with access to the allocation sequence and was not involved in participant recruitment or eligibility assessment. After the nurses conducting the home visits had verified the inclusion and exclusion criteria and completed the baseline assessment, they contacted the fieldwork coordinator, who opened the next sequentially numbered envelope and communicated the corresponding group assignment. Thus, personnel responsible for recruitment and eligibility assessment had no access to the allocation sequence and could not anticipate upcoming assignments.

The sample size calculation was based on the primary outcome, the number of falls during the 12-month follow-up. Based on previous evidence, a fall rate of 1.5 falls per person-year was assumed for the control group [17]. The intervention was expected to reduce this rate to 1 fall per person-year, corresponding to an incidence rate ratio (IRR) of approximately 0.67. Assuming a two-sided significance level of 0.05, 80% statistical power, and allowance for overdispersion in fall counts, a minimum sample of 200 participants (100 per group) was estimated. To account for an anticipated 10% attrition rate, the target sample was increased to 220 participants (110 per group). The participant recruitment and allocation process is presented in Figure 1.

### 2.3. PM ACTIVAS Intervention

Participants allocated to the intervention group received the PM ACTIVAS model over a 12-month period in addition to usual primary care. Intervention delivery was undertaken by recently graduated nurses and final-year nursing students who completed the standardized PM ACTIVAS training program before delivering the intervention.

Because the PM ACTIVAS intervention has been fully described elsewhere according to the TIDieR checklist, only the intervention components necessary to interpret the trial results are summarized below [17].

Briefly, the PM ACTIVAS nurse-led preventive care model operationalizes evidence-based fall prevention by integrating:

○ Multidimensional assessment of intrinsic and extrinsic fall risk factors;
○ Individualized risk management and shared care planning;
○ Home environmental assessment and modification;
○ Individualized education and counselling;
○ Provision of a home safety kit;
○ Family involvement whenever appropriate; and
○ Structured monthly telephone follow-up to reinforce agreed preventive actions.

The intervention is grounded in person-centred care and seeks to operationalize individualized evidence-based fall prevention by translating multidimensional risk assessment into personalized behavioural and environmental strategies that support healthy ageing and ageing in place.

Intervention delivery was standardized through a structured training and implementation process. All interventionists completed a 20 h synchronous online theoretical and practical training course on the PM ACTIVAS fall-prevention model and received a standardized intervention manual. Additional implementation materials included home-visit and telephone follow-up protocols and standardized registration forms.

Intervention fidelity was supported through direct supervision of each interventionist’s initial home visits and telephone follow-ups by the project coordinator, ongoing review of activity records and follow-up forms, and regular individual and group monitoring meetings to address implementation issues and reinforce adherence to the intervention protocol. One of the principal investigators provided ongoing oversight of the implementation process.

Participants allocated to the control group received usual primary care, including the Examen de Medicina Preventiva del Adulto Mayor (EMPAM), the preventive health assessment routinely provided to older adults in Chilean primary health care. The EMPAM assesses overall health and functional status and includes assessment of fall-related risk. However, usual care did not include the specific components of the PM ACTIVAS model, including structured multidimensional assessment of intrinsic and extrinsic fall-risk factors, systematic home environmental assessment, individualized fall-prevention planning developed with the older adult and family, or structured monthly telephone follow-up. Both groups completed a Falls and Events Calendar throughout the follow-up period [17].

### 2.4. Outcome Measures

#### 2.4.1. Primary Outcome

The primary outcome was the total number of falls recorded during the 12-month follow-up period. Participants in both the intervention and control groups prospectively recorded falls using a monthly Falls and Events Calendar, which constituted the principal outcome measure of the randomized trial. The calendar was provided at recruitment, and participants were instructed to record the days on which a fall, trip, or related event occurred. Family members or caregivers living in the same household and involved in the study could also assist with the recording process. Both participants and caregivers were instructed to use a standardized definition of a fall as any event resulting in the person coming into contact with the ground, regardless of its severity or consequences.

To enhance the completeness and reliability of prospective reporting, participants were contacted by telephone once a month throughout the 12-month follow-up period. During these contacts, the research team emphasized the importance of keeping the Falls and Events Calendar complete and up to date and addressed any questions regarding its completion. When appropriate, contact could also be established with a family member who lived with the participant and was familiar with the study activities. The completed calendars were collected by the research team during the final home visit.

Falls occurring during the 12 months preceding enrollment were also retrospectively reported at baseline. However, these data were used for baseline characterization rather than to assess change in fall occurrence. Unlike the prospective follow-up, baseline reports were not based on systematic monitoring using the study calendar or a standardized definition of a fall and were subject to recall bias, particularly given the 12-month recall period in an older population. Therefore, baseline and follow-up fall counts were not considered directly comparable, and change in the number of falls from baseline to follow-up was not used as an outcome. Instead, the primary outcome was based exclusively on prospectively recorded falls during the 12-month follow-up period.

#### 2.4.2. Secondary Outcomes

Secondary outcomes included fall occurrence (≥1 fall during follow-up); intrinsic and extrinsic fall-risk factors; ability to identify environmental fall hazards; and fear of falling. Fall-risk factors were assessed using a 37-item structured clinical checklist comprising nine intrinsic risk factors (e.g., medication use and muscle stiffness) and 28 extrinsic risk factors (e.g., uneven surfaces in the home and inappropriate footwear). Each factor was recorded dichotomously as present or absent. The checklist was designed to identify fall-risk factors recognized in the evidence and to support individualized identification of potentially modifiable risks; it was not intended as a predictive scale to estimate an individual’s probability of falling. The number of identified risk factors was summed, yielding a total count ranging from 0 to 37, with higher values indicating a greater number of identified risk factors rather than an estimated level of fall risk.

Environmental fall-hazard recognition was assessed using an illustrated tool comprising four household scenarios depicting potential hazards in different areas of the home. Participants were asked to identify fall hazards represented in each scenario, with higher scores indicating greater hazard recognition (Supplementary Table S4). Earlier versions of both instruments were used in a randomized controlled trial conducted by the same research team among community-dwelling older adults in Chile [10], and their development, content, and scoring procedures have been described in the PM ACTIVAS study protocol [17].

### 2.5. Data Collection

Baseline assessments were conducted during home visits after confirming eligibility criteria and obtaining written informed consent. Data collection included a structured questionnaire composed of six modules, administration of study instruments, and blood pressure measurement. During the COVID-19 pandemic, recruitment and data collection followed institutional infection prevention measures, including personal protective equipment, daily symptom screening, and SARS-CoV-2 testing when required.

Follow-up assessments were completed 12 months after baseline. Falls were prospectively monitored using the monthly Falls and Events Calendar throughout the study period.

### 2.6. Data Analysis

Baseline comparability between the intervention and control groups was assessed using Mann–Whitney U tests for continuous variables and chi-square tests for categorical variables. The Mann–Whitney U test was selected based on the results of the Kolmogorov–Smirnov normality test, which indicated significant departures from normality for all continuous variables analyzed (Supplementary Table S2). All analyses followed the intention-to-treat principle, with participants analyzed according to their originally assigned group. No imputation procedures were applied for missing data; instead, analyses were conducted using an available-case approach, including all participants with available data for each outcome. Although 205 participants completed the 12-month follow-up, complete primary-outcome data on the number of falls during follow-up were available for 145 participants because some self-reported monthly Falls and Events Calendars were incomplete. Accordingly, analyses of the primary outcome included all participants with available fall data. Comparisons of baseline characteristics between participants with and without available primary-outcome data are presented in Supplementary Table S5.

The primary outcome, the number of falls during the 12-month follow-up, was analyzed as a count outcome using negative binomial regression, with intervention group as the main predictor. A sensitivity analysis was conducted to account for baseline fall history by additionally including the number of falls reported during the 12 months preceding enrollment as a covariate in the model.

For secondary outcomes, fall occurrence during the 12-month follow-up was dichotomized as having experienced at least one fall versus no falls and compared between the intervention and control groups using the chi-square test. A sensitivity analysis was conducted using binary logistic regression, with fall occurrence during follow-up as the dependent variable and intervention group and baseline fall occurrence (i.e., at least one fall during the 12 months preceding enrollment) as predictors. For fall-risk factors and home-hazard recognition, analysis of covariance (ANCOVA) was performed, with the follow-up value as the dependent variable, intervention group as the fixed factor, and the corresponding baseline score as a covariate. Adjusted effect sizes (partial η^2^) were calculated to facilitate interpretation of the magnitude of between-group differences (Supplementary Table S3). For fear of falling, an analogous baseline-adjusted approach was applied using binary logistic regression, with fear of falling at follow-up as the dependent variable and intervention group and baseline fear of falling as predictors. Statistical significance for the primary outcome was set at *p* < 0.05, whereas secondary outcome analyses were considered complementary and interpreted accordingly.

### 2.7. Ethical Considerations

The study was conducted in accordance with the Declaration of Helsinki and approved by the Institutional Ethics Committee (Approval No. 190318014; 20 December 2019). The trial was prospectively registered at ClinicalTrials.gov (https://clinicaltrials.gov/study/NCT04313062; accessed on 16 March 2020). Written informed consent was obtained from all participants before enrolment.

Generative artificial intelligence was not used during the design of the study, participant recruitment, data collection, statistical analysis, or interpretation of findings. Any AI-assisted support used during manuscript preparation was limited to editorial writing assistance and will be reported in the Acknowledgments section in accordance with the journal’s policy.

## 3. Results

### 3.1. Participant Flow and Baseline Characteristics

A total of 220 community-dwelling older adults were randomized to either the PM ACTIVAS intervention group (n = 110) or the control group receiving usual primary care (n = 110). Of the 205 participants who completed the 12-month follow-up, complete data on the primary outcome (number of falls during follow-up) were available for 145 participants because some self-reported monthly fall calendars contained incomplete information (Figure 1).

No statistically significant baseline differences were observed between the intervention and control groups for the characteristics examined (Table 1).

Comparisons between participants with and without available primary-outcome data showed few baseline differences; within the intervention group, significant differences were observed only for age and home-hazard recognition, whereas no other baseline characteristics differed significantly. No significant baseline differences according to primary-outcome data availability were observed within the control group (Supplementary Table S5).

### 3.2. Primary Outcome

During the 12-month follow-up, no statistically significant between-group difference was observed in the number of falls, the primary outcome (*p* = 0.164) (Table 2). A sensitivity analysis using negative binomial regression and adjusting for the number of falls reported during the 12 months preceding enrollment yielded similar findings. No significant intervention effect on the number of falls during follow-up was observed after adjustment for the number of falls reported at baseline (IRR = 0.65, 95% CI [0.34, 1.26]). Baseline number of falls was also not significantly associated with the rate of falls during follow-up (IRR = 1.04, 95% CI [0.88, 1.38]). These findings indicate that adjustment for baseline fall history did not materially alter the primary outcome results.

### 3.3. Secondary Outcomes

#### 3.3.1. Changes in Fall Occurence

Regarding fall occurrence, a secondary outcome, falls were reported by 17 participants (23.3%) in the intervention group and 19 participants (26.4%) in the control group. No statistically significant between-group difference was observed (*p* = 0.666; Table 2). Sensitivity analyses adjusting for falls reported during the 12 months preceding enrollment yielded similar findings. In the logistic regression model, baseline fall occurrence was significantly associated with fall occurrence during follow-up (OR = 2.62, 95% CI [1.19, 5.80]); however, no significant intervention effect was observed after adjustment for baseline fall occurrence (OR = 1.05, 95% CI [0.48, 2.30]) (Table 2).

#### 3.3.2. Changes in Fall-Related Risk Factors

At the end of follow-up, participants receiving the PM ACTIVAS intervention had significantly fewer modifiable fall-risk factors than those receiving usual care, after adjustment for the baseline number identified fall-risk factors (*p* = 0.001), with a moderate effect size (partial η^2^ = 0.07) (Table 2). The adjusted between-group mean difference was −1.42 identified risk factors (95% CI [−2.17, −0.67]), indicating that participants in the intervention group had, on average, 1.42 fewer identified modifiable fall-risk factors than those in the control group.

#### 3.3.3. Recognition of Environmental Fall Hazards

After adjustment for the baseline number of identified fall-risk factors, participants receiving the PM ACTIVAS intervention demonstrated significantly greater recognition of environmental fall hazards than those receiving usual care (*p* = 0.044), although the effect size was small (partial η^2^ = 0.02) (Table 2). The adjusted between-group difference was 0.72 points (95% CI [0.02, 1.41]), favoring the intervention group. This finding should be interpreted with caution, as participants in the intervention group with available follow-up data for this outcome had baseline scores approximately 1 point higher than those without available follow-up data, and this difference was statistically significant (*p* = 0.04).

#### 3.3.4. Fear of Falling

No statistically significant differences between groups were observed regarding fear of falling after the intervention (Table 2).

## 4. Discussion

This study evaluated the clinical effects of the PM ACTIVAS nurse-led preventive care model among community-dwelling older adults within routine primary health care. Although the PM ACTIVAS nurse-led preventive care model did not significantly reduce the number of falls over 12 months, it was associated with statistically significant but modest improvements in modifiable fall-risk factors and environmental hazard recognition. These findings suggest that an important contribution of PM ACTIVAS lies not only in its evidence-based intervention components but also in the way evidence-based fall prevention was operationalized through a coordinated nurse-led preventive care model. This interpretation aligns with recent evidence indicating that the effectiveness of multifactorial interventions depends not only on the combination of intervention components but also on their individualized application, coordination, and integration into routine care [11,21].

The observed reduction in fall-risk factors may reflect the synergistic integration of multiple evidence-based strategies rather than by the effect of any single intervention component [22]. PM ACTIVAS combines multidimensional risk assessment, individualized education, home-hazard identification, tailored recommendations, and longitudinal follow-up within the PM ACTIVAS nurse-led preventive care model. This approach is consistent with recent evidence indicating that the effectiveness of fall prevention programmes depends on combining complementary intervention components that address both intrinsic and extrinsic risk factors while adapting preventive strategies to individual needs and contexts [23,24,25]. Furthermore, implementation research has demonstrated that successful community-based fall prevention requires more than evidence-based interventions alone; it also depends on effective coordination, patient engagement, adaptation to local settings, and sustained professional support throughout the implementation process [26,27]. Together, these findings suggest that the observed changes in intermediate outcomes may be attributable not only to the evidence-based intervention components but also to the structured nurse-led preventive care model through which those components were coordinated and integrated into routine primary health care. This may help explain why multifactorial interventions with similar components often demonstrate heterogeneous effectiveness across studies.

The improvement observed in these intermediate outcomes did not translate into a statistically significant reduction in the number of falls during the 12-month follow-up. This discrepancy is plausible because falls are distal and multifactorial events that result from the interaction of multiple intrinsic, behavioral, environmental, and time-varying factors, not all of which were directly modified or measured in this study. Moreover, the magnitude of the observed changes in the secondary outcomes was modest: participants in the intervention group had an adjusted mean of 1.42 fewer identified fall-risk factors than controls (partial η^2^ = 0.07), while the improvement in environmental hazard recognition was 0.72 points (partial η^2^ = 0.02). Such changes may represent progress in potentially modifiable determinants of falls without necessarily being sufficient to produce a detectable reduction in fall events over the study period. In addition, complete primary-outcome data were available for 145 of the 220 randomized participants, reducing the precision of the treatment-effect estimate. Although the adjusted incidence rate ratio was 0.65, its wide 95% confidence interval (0.34–1.26) included both a potentially meaningful reduction and no intervention effect; therefore, the present findings should not be interpreted as evidence of a trend toward benefit. Overall, these results suggest that improvements in selected intermediate fall-prevention outcomes should be distinguished from demonstrated effectiveness in reducing falls themselves.

Our findings complement the current international evidence on community-based fall prevention by addressing an important gap between intervention efficacy and real-world implementation. Recent systematic reviews and clinical recommendations consistently conclude that multifactorial interventions can reduce fall risk among community-dwelling older adults, although their overall effects are often modest and heterogeneous across populations and intervention designs [7,11]. Rather than contradicting these findings, the PM ACTIVAS nurse-led preventive care model provides a structured framework for operationalizing evidence-based fall prevention within routine primary health care. This implementation-oriented perspective aligns with recent calls to move beyond evaluating individual intervention components toward understanding how evidence-based strategies can be effectively delivered, coordinated, and sustained in real-world community settings [19,23,26]. Consequently, the contribution of the present study includes not only in the observed reductions in modifiable fall-risk factors but also the illustration of how evidence-based fall prevention can be systematically integrated into routine primary health care through coordinated nursing practice. Accordingly, the innovation of PM ACTIVAS resides less in the individual intervention components than in the structured nurse-led model through which those components are coordinated, individualized, and embedded within routine primary health care [28].

The implications of these findings extend beyond fall prevention itself. Falls are recognized as one of the leading causes of functional decline, loss of independence, and increased health care utilization among older adults, making their prevention a priority for primary health care systems facing rapid population ageing [3]. In this context, PM ACTIVAS illustrates how nurses can lead a coordinated preventive care delivery model by integrating comprehensive assessment, individualized care planning, health education, home-based interventions, and longitudinal follow-up into routine practice [29,30,31]. This approach is consistent with recent clinical recommendations emphasizing that fall prevention should be embedded within comprehensive, person-centered care rather than delivered as isolated clinical activities [5,7,32]. By strengthening continuity of care and proactively addressing modifiable risk factors, nurse-led preventive models such as PM ACTIVAS may provide a structured approach to integrating fall-prevention strategies into routine care while supporting broader healthy-ageing goals [2].

This study has several strengths that enhance the relevance of its findings. First, it evaluated a comprehensive nurse-led preventive care model implemented under routine primary health care conditions, supporting the relevance of the findings to real-world primary health care practice. Second, the multidimensional assessment of intrinsic and extrinsic fall-risk factors enabled the evaluation of clinically meaningful changes beyond the occurrence of falls alone, capturing changes in identified modifiable risk factors that constitute relevant intermediate outcomes in fall prevention [27]. Nevertheless, several limitations should be acknowledged. The study was conducted within a single primary health care setting, which may limit the generalizability of the findings to other populations and health systems. Although overall retention at 12 months was high, incomplete self-reported monthly Falls and Events Calendars resulted in missing primary-outcome data, reducing the sample available for the primary effectiveness analysis and potentially limiting statistical power and precision to detect between-group differences in the number of falls.

In addition, falls were self-reported by participants and may therefore have been subject to reporting bias. Although both groups used the same monthly Falls and Events Calendar, participants in the intervention group had greater exposure to fall-prevention assessment, education, and longitudinal follow-up, which may have increased their awareness and recognition of fall events. Consequently, differential reporting between groups cannot be excluded, although its presence, direction, and magnitude could not be determined in this study. Footwear type and appropriateness were not systematically assessed as study variables and therefore could not be included in the analysis. Given that inappropriate footwear and foot-related factors are recognized contributors to fall risk among community-dwelling older adults, this represents a potential unmeasured confounder that may have influenced fall outcomes [4,5].

Missing follow-up data may also have influenced the observed effect on environmental hazard recognition. Specifically, within the intervention group, participants with available follow-up data had significantly higher baseline hazard-recognition scores than those without follow-up data, indicating some baseline differences associated with data availability that should be considered when interpreting this finding. Therefore, the small improvement observed for this secondary outcome should be interpreted with caution. Furthermore, while the study evaluated fall outcomes, it did not examine other clinically important outcomes, including fall-related injuries, hospitalizations, or health-related quality of life. Future multicenter studies using standardized prospective outcome collection with longer follow-up periods are needed to determine whether changes in modifiable fall-risk factors translate into sustained reductions in falls and other clinically important outcomes across diverse primary health care settings, and to assess the model’s transferability and scalability.

## 5. Conclusions

The PM ACTIVAS nurse-led preventive care model did not significantly reduce the number of falls among community-dwelling older adults during the 12-month follow-up. However, the intervention was associated with fewer identified modifiable fall-risk factors and improved environmental hazard recognition, suggesting effects on selected intermediate outcomes of the fall-prevention process.

By integrating evidence-based fall-prevention strategies within a coordinated nurse-led care pathway, PM ACTIVAS provides a structured model for implementing preventive care in routine primary health care. Further adequately powered studies with complete prospective ascertainment of falls are needed to determine whether improvements in these intermediate outcomes can translate into reductions in falls and other clinically important outcomes.

## Figures and Tables

**Figure 1:**
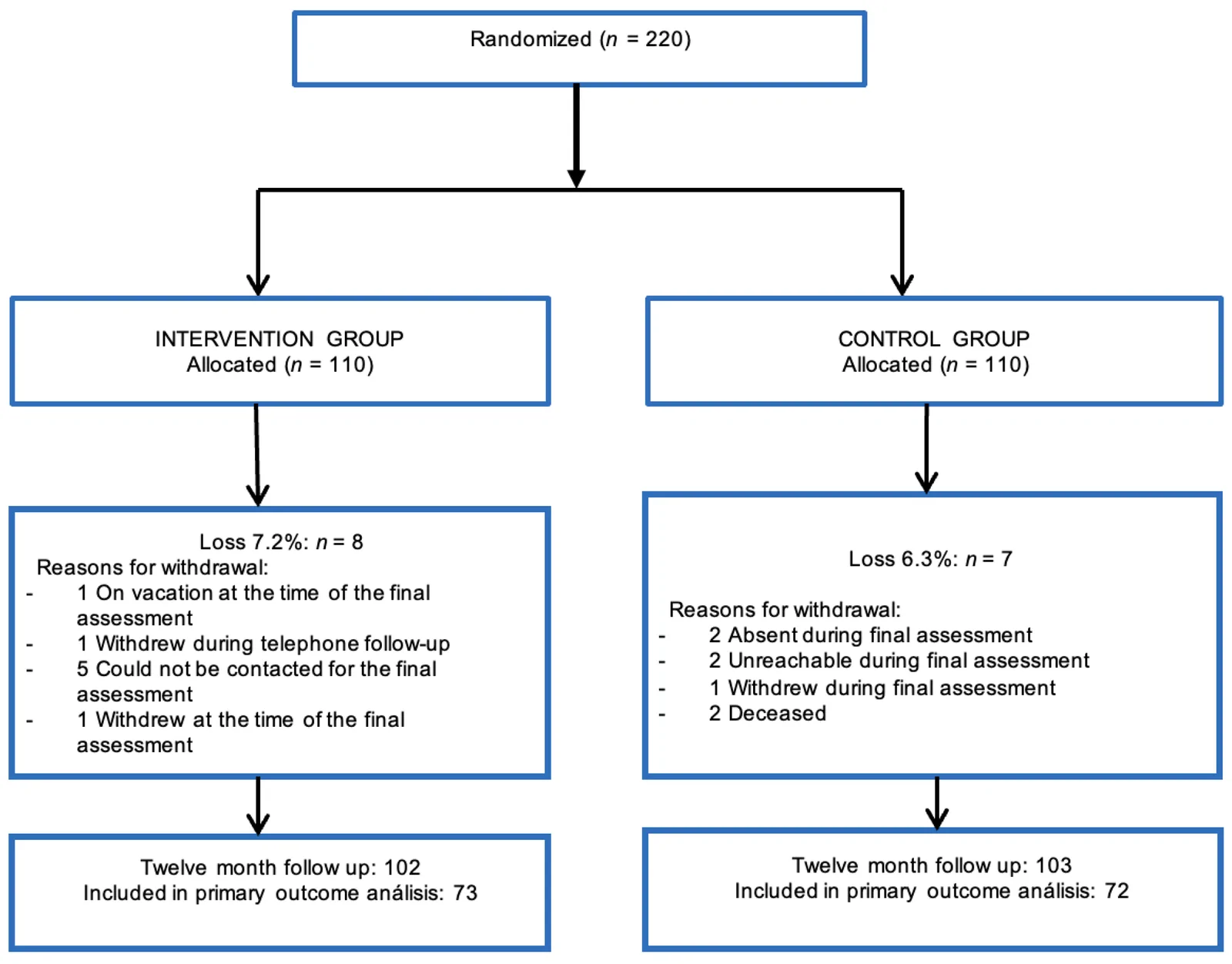
CONSORT flow diagram of participant recruitment, allocation, follow-up, and primary-outcome analysis.

**Table 1:** Baseline characteristics of study participants according to study group.

Characteristic	Intervention (n = 110)	Control (n = 110)	Test Statistic (U/χ^2^)	*p* Value
Age, years, median (IQR)	72 (15)	72 (13)	6004.5	0.957 ^a^
Female sex, n (%)	94 (85.5)	100 (90.9)	1.57	0.210 ^b^
Education (years), median (IQR)	8 (16)	8 (17)	6609.0	0.147 ^a^
MMSE, median (IQR)	18 (6)	18 (6)	6375.0	0.471 ^a^
EFAM-A, median (IQR)	49 (11)	50 (11)	6877.0	0.078 ^a^
EFAM-B, median (IQR)	43 (43)	44 (41)	6505.0	0.335 ^a^
Previous falls, n (%)	37 (34.3)	39 (36.1)	0.08	0.776 ^b^
Fear of falling, n (%)	82 (74.5)	80 (73.4)	0.04	0.846 ^b^
Identified fall-risk factors, median (IQR)	13 (14)	12 (16)	5273.0	0.222 ^a^
Home-hazard recognition score, median (IQR)	11 (15)	11 (16)	6483.0	0.294 ^a^

IQR: interquartile range. ^a^ Statistics based on the Mann–Whitney U test. ^b^ Statistics based on the chi-square test. MMSE: Mini-Mental State Examination. EFAM-A/EFAM-B: Evaluación Funcional del Adulto Mayor (Functional Assessment of the Older Adult), Parts A and B.

**Table 2:** Primary and secondary outcomes at baseline and 12-month follow-up.

Outcome	Intervention Baseline	Intervention Follow-Up	Control Baseline	Control Follow-Up	Between-Group *p*
Primary outcome					
Number of falls, median (IQR)	0 (4) ^a^	1 (5)	0 (8) ^a^	2 (5)	0.164
Secondary outcomes					
Fall occurrence, n (%)	37 (34.3) ^a^	17 (23.3)	39 (36.1) ^a^	19 (26.4)	0.666
Fall-risk factors, median (IQR)	13 (14)	10 (15)	12 (16)	11 (14)	0.001
Home-hazard recognition, median (IQR)	11 (15)	12 (12)	11 (16)	11.5 (13)	0.044
Fear of falling, n (%)	82 (74.5)	29 (28.4)	80 (73.4)	29 (28.2)	0.558

^a^: Indicates a self-reported measure; these measures were included in the sensitivity analyses. *p* values correspond to the between-group comparisons described in Section 2.6 (chi-square test, negative binomial regression, ANCOVA, or logistic regression, as appropriate).

## Data Availability

The datasets generated and analyzed during the current study are not publicly available because they contain potentially identifiable participant information but are available from the corresponding author on reasonable request and subject to approval by the Institutional Ethics Committee.

## Supplementary Materials

The following supporting information can be downloaded at https://www.mdpi.com/article/10.3390/healthcare14193151/s1: Table S1: CONSORT 2025 Checklist; Table S2: Normality testing for continuous variables at baseline; Table S3: Estimated marginal means for the number of identified fall-risk factors at 12-month follow-up; Table S4: Estimated marginal means for home-hazard recognition at 12-month follow-up; Table S5: Baseline characteristics of study participants according to study group and primary-outcome data availability.
