# Supplementary material for: Evaluating the PM ACTIVAS Nurse-Led Preventive Care Model for Community-Dwelling Older Adults: A Randomized Controlled Trial
Source: Healthcare (Basel). 2026 Sep 23;14(19):3151. doi: 10.3390/healthcare14193151 (PMC13665406; doi:10.3390/healthcare14193151)
Supplement: Supplementary file 1 [file healthcare-14-03151-s001.zip › healthcare-4515957-supplementary3.pdf]

Supplementary Material

Table S2. Normality test (Kolmogorov–Smirnov) for continuous variables at baseline.

Variable	Statistic	df	Sig.
Age	0.083	213	0.001
Education, years	0.149	213	<0.001
MMSE	0.245	213	<0.001
EFAM-A	0.136	213	<0.001
EFAM-B	0.091	213	<0.001
Fall-risk checklist score	0.087	213	<0.001
Home hazard recognition score	0.146	213	<0.001

Kolmogorov–Smirnov test with Lilliefors significance correction. Sig. < 0.05 indicates a significant deviation from a normal distribution; non-parametric (Mann–Whitney U) tests were therefore used for between-group comparisons of continuous variables.

Table S3. Estimated marginal means for the number of identified fall-risk factors at 12-month follow-up (ANCOVA, adjusted for baseline value)

Group	Mean	SE	95% CI lower	95% CI upper
Intervention	9.659	0.270	9.127	10.190
Control	11.080	0.268	10.551	11.610

Estimated marginal means adjusted for the baseline number of identified fall-risk factors (12.4826).

Table S4. Estimated marginal means for the home hazard recognition score at 12-month follow-up (ANCOVA, adjusted for baseline score).

Group	Mean	SE	95% CI lower	95% CI upper
Intervention	11.524	0.250	11.032	12.017
Control	10.809	0.250	10.317	11.302

Estimated marginal means adjusted for the covariate in the model: baseline home hazard recognition score = 10.5686.

Table S5. Baseline characteristics of study participants according to study group and follow-up data availability.

Characteristic	Intervention–with data (n=73)	Intervention–without data (n=37)	p	Control–with data (n=72)	Control–without data (n=38)	p
Age, years, median (range)	72 (14)	74 (15)	0.012[a]	72 (13)	71 (13)	0.137[a]
Female sex, n (%)	63 (86.3)	31 (83.8)	0.723[b]	65 (90.3)	35 (92.1)	0.751[b]
Education, years, median (range)	8 (15)	8 (14)	0.182[a]	8 (17)	8.5 (14)	0.253[a]
MMSE, median (range)	18 (6)	18 (6)	0.629[a]	18 (6)	18 (4)	0.545[a]
EFAM-A, median (range)	49 (11)	49 (11)	0.896[a]	50 (11)	50.5 (11)	0.942[a]
EFAM-B, median (range)	43 (42)	42 (41)	0.502[a]	44.5 (41)	43.5 (36)	0.563[a]

Characteristic	Intervention–with data (n=73)	Intervention–without data (n=37)	p	Control–with data (n=72)	Control–without data (n=38)	p
Home hazard recognition score, median (range)	11 (14)	10 (11)	0.040[a]	12 (16)	10 (14)	0.065[a]
Fall-risk checklist score, median (range)	13 (14)	14 (13)	0.188[a]	12 (16)	13 (11)	0.063[a]
Fear of falling, n (%)	54 (74.0)	28 (75.7)	0.846[b]	51 (71.8)	29 (76.3)	0.614[b]
Number of previous falls, median (range)	0 (4)	0 (3)	0.268[a]	0 (4)	0 (8)	0.501[a]

Data are presented as median (range) for continuous variables and n (%) for categorical variables. Groups were defined by allocated treatment (intervention/control) and by availability of the primary outcome at the 12-month follow-up (with data/without data). p values compare with-data vs. without-data subgroups within each treatment arm. [a] Statistics based on the Mann–Whitney U test. [b] Statistics based on the chi-square test. MMSE: Mini-Mental State Examination. EFAM-A/EFAM-B: Evaluación Funcional del Adulto Mayor (Functional Assessment of the Older Adult), Parts A and B.
